# Differentiation of Insect Flours by Elemental Analysis and Chemometrics: A Study Using Inductively Coupled Plasma Mass Spectrometry (ICP-MS)

**DOI:** 10.3390/molecules29245878

**Published:** 2024-12-12

**Authors:** Mattia Montanaro, Alessandra Biancolillo, Angelo Antonio D’Archivio, Martina Foschi

**Affiliations:** Department of Physical and Chemical Sciences, University of L’Aquila, Via Vetoio snc, 67100 L’Aquila, Italy; mattia.montanaro@student.univaq.it (M.M.); angeloantonio.darchivio@univaq.it (A.A.D.); martina.foschi@univaq.it (M.F.)

**Keywords:** insect flour, cricket flour, ICP-MS, explorative analysis, PCA, discriminant classification, LDA, variable selection, covariance selection

## Abstract

Background: This study aimed to validate a method for characterizing and quantifying the multi-elemental profiles of different insect flours to enable their distinction, identification, and quality assessment. The focus was on three insect species: cricket (*Acheta domesticus*), buffalo worm (*Alphitobius diaperinus*), and mealworm (*Tenebrio molitor*). Methods: Mealworms were powdered in the laboratory through mechanical processing. Sample analysis involved acid digestion using a microwave digester, followed by profiling with Inductively Coupled Plasma Mass Spectrometry (ICP-MS). This technique enabled rapid, multi-elemental analysis at trace levels. Chemometric methods, including Principal Component Analysis (PCA) for exploratory analysis, Covariance Selection-Linear Discriminant Analysis (CovSel-LDA), alongside forward stepwise LDA classification methods, were applied and compared. Results: ICP-MS accurately detected elements at micro trace levels. Both classification models, based on different variable selection methods and externally validated on a test set comprising 45% of the available samples, proved effective in classifying samples based on slightly different pools of trace elements. CovSel-LDA selected Mg and Se, whereas the stepwise-LDA focused on Mg, K, and Mn. Conclusions: the validated methods demonstrated high accuracy and generalizability, supporting their potential use in food industry applications. This model could assist in quality control, facilitating the introduction of insect-based flour into European and international markets as novel foods.

## 1. Introduction

In the context of alternative food sources, edible insect flours are playing an increasingly central role in addressing the challenges associated with sustainable nutrition. Entomophagy has become quite widespread, as evidenced by information from the FAO (Food and Agriculture Organization of the United Nations): according to their data, over 2 billion people consume more than 2000 species of insects [1]. Although this practice is still relatively uncommon in Europe, this practice is traditional in parts of Asia, South America, and Africa [2]. The consumption of edible insects, and especially of their derivatives such as flour, can address various environmental and economic issues. According to the United Nations Educational, Scientific and Cultural Organization (UNESCO) data, the world population increase is leading to a depletion of available resources; in this context, the vast availability of edible insects in nature could be a compelling reason to promote their production and consumption on a large scale [3].

Fiebelkorn and collaborators have shown that in vitro insect farming enables a reduction in CO_2_ emissions, lower water demand, and decreased land use; due to their sustainability, many companies have already recognized the economic potential of edible insects [4]. However, most studies on edible insects focus on their nutritional profile, which is also highly diverse [5]. Many edible insects can meet the amino acid requirements of the human diet; in addition to proteins, they are also rich in polyunsaturated fatty acids, contain high levels of essential minerals (copper, iron, magnesium, manganese, phosphorus, selenium, and zinc), and include fibers, such as chitin, not found in traditional meats [6]. Insects exhibit a highly variable protein content, ranging from 13% to 77%, depending on the development stage and order [6]. Edible insects are rich in oleic, linoleic, and linolenic acids, though their fatty acid profile depends heavily on their diet. Mineral content in insects is also extremely variable; not all edible insects can meet daily mineral requirements [7]. However, it has been shown that in some cases, certain mineral contents in insects can exceed those found in traditional meats; for example, beef has an average zinc content of approximately 12.5 mg per 100 g of dry weight, while *R. phoenicis* larvae contain 26.5 mg per 100 g [6].

While most studies focus on the protein and fatty acid profiles in insect flours, there are not numerous examples in the literature where flours can be characterized by assessing their multi-element profile, which, as noted above, is nutritionally significant. Indeed, some insect flours have been analyzed by infrared spectroscopy (IR). Benes and collaborators [8] have shown that it is possible to accurately differentiate and identify flour from seven insect species mixed with wheat flour; this study employed near-infrared spectrophotometry (NIR) coupled with chemometric classification methods. Another study [9], conducted by Foschi et al., aimed to use an IR spectrometer to establish a procedure for detecting potential adulterations in cricket flour through spectral comparison. Using Inductively Coupled Plasma Mass Spectrometry (ICP-MS) in studies on insect flours represents an innovative and effective alternative for their classification. The originality of this approach is evidenced by the fact that it is commonly applied to plant-based flours, such as rice flour [10], while studies on insect flours remain relatively limited. Its effectiveness, on the other hand, stems from its significant advantages in terms of precision and sensitivity. In light of this, the present study aims at validating a method for characterizing and quantifying the multi-elemental profiles of different insect flours to enable their distinction, identification, and quality assessment. The focus was on three insect species: cricket (*Acheta domesticus*), buffalo worm (*Alphitobius diaperinus*), and mealworm (*Tenebrio molitor*). This exploits the sensitivity of ICP-MS, coupled with advanced chemometric methods, to provide a robust framework for the classification and quality assessment of insect flours. By identifying trace elements critical for discrimination, this research represents a novel application of ICP-MS to a burgeoning area of sustainable food science.

## 2. Results and Discussion

### 2.1. ICP-MS Analysis and Validation

The ICP-MS analysis has been run as described in Section 3.2.

Table 1 presents the isotopes of the elements analyzed, the lower and upper concentrations, expressed in µg/L, of the respective calibration curves, and the coefficients of determination (R^2^).

The calibration curves and independent measurements of blank samples were employed to determine the limit of detection (LOD) and the limit of quantification (LOQ). Table 2 presents the validation parameters assessed according to Eurachem guidelines [11]. Specifically, it includes the relative standard deviation (RSD) of the method, estimated from five replicates of unfortified samples, the recovery rates for two matrices (crickets and buffalo worms), also calculated from five replicates for each class, as well as the LOD and LOQ expressed in µg/g of dried samples.

The average concentrations (expressed in µg/g of dried sample) of the quantified elements in the different classes, based on 24 samples for crickets (C) and buffalo worms (B) and 28 samples for mealworms (W), are reported in Table 2.

The results obtained in this study are generally in agreement with those reported by Sikora et al. [12], considering that they mainly analyzed insect-based products (i.e., insect flours mixed with other food ingredients), while our study focuses specifically on pure insect flours. The RSD values for all quantifiable analytes fall within an acceptable range, indicating the method’s robustness [13]. Notably, boron could not be quantified across all tested classes. For other elements, including chromium (Cr), selenium (Se), and strontium (Sr), accurate quantification was not achievable in some of the three classes. Furthermore, nickel (Ni), selenium (Se), and cadmium (Cd) were found close to the quantification limits; however, it was demonstrated that this low concentration did not lead to unacceptable RSD or recovery values for Cd and Ni.

Regarding recoveries, they were found to be close to 100%, with few exceptions (such as Co, which is slightly above the accepted limits), and they were very similar between the two matrices (crickets and buffalo worms), indicating both the accuracy and stability of the method. Based on RSD values and recovery rates, this method can be deemed acceptable in terms of precision and accuracy.

### 2.2. Explorative Analysis

After preprocessing data by autoscaling, Principal Component Analysis was used to assess potential patterns or outliers in the analyzed flour samples. The Principal Component Analysis (PCA) model enables the creation of biplots, i.e., a graphical representation of the projection of samples and variables onto the space of the first two principal components (PCs). From the plot, it is possible to derive that cricket flour (yellow dots) is richer in elements such as Mn, Zn, and Se, with respect to the other two classes. In contrast, elements like Mg, Cd, and Mo are in higher quantities in mealworm flour. The second principal component is dominated by other elements, such as K, found in greater quantities in buffalo worm flour (green downward triangle), or Sr and Ba, which are prevalent in mealworms. In general, mealworms are richer in heavy metals like Cd and Mo but poor in essential minerals, which are abundant in buffalo worms and crickets. This may stem from the fact that the analyzed mealworms come from a sample not intended for human consumption, unlike cricket and buffalo worm flour. The exploratory analysis was conducted using all available variables without any prior selection based on method validation parameters or statistical analysis. The variables, represented as black dots, are labeled according to the instrumental analysis mode and the specific isotope.

### 2.3. Classification

**Figure 1:**
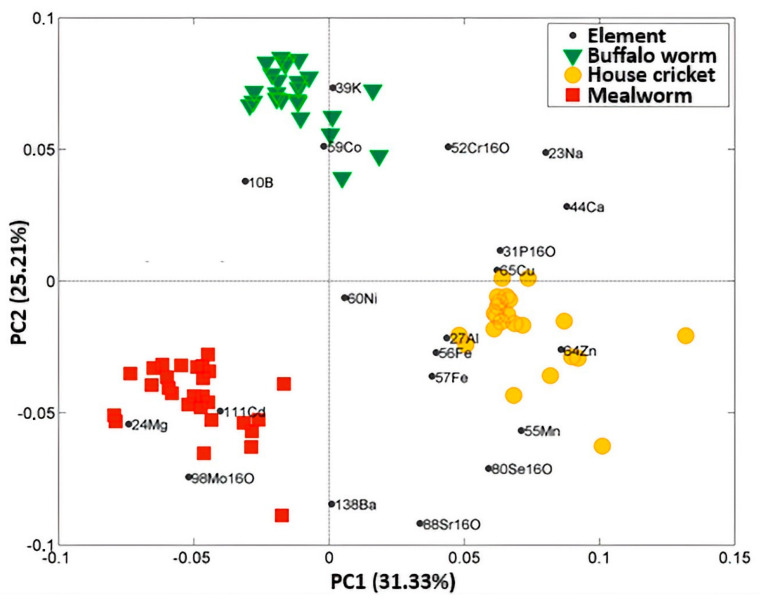
Biplot with the first two principal components. The percentage in brackets is the variance explained by each PC. Legend: Black dots represent the diverse quantified elements. Green downward triangles depict buffalo worms; yellow circles for house crickets; and red squares represent mealworms.

**Figure 2:**
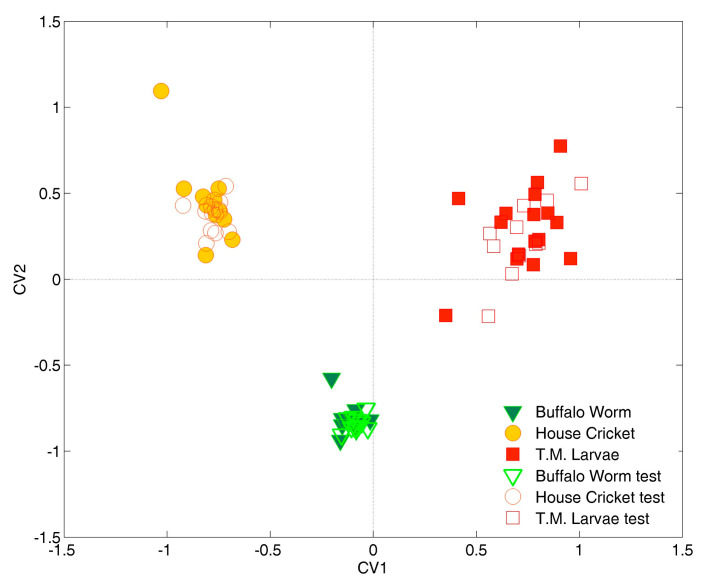
Projection of samples (filled and empty symbols refer to training and test samples, respectively) onto the canonical variates (CVs) obtained by the CovSel-LDA model.

A cross-validation procedure was combined with two different variable selection methods: Covariance Selection (CovSel) and Forward Stepwise Selection based on Wilks’ lambda statistics. To determine the optimal number of original variables using CovSel, a 7-fold cross-validation procedure was applied. Through this cross-validation, the optimal variables identified were magnesium (Mg) and selenium (Se). The orthogonality of the vector rays of Mg and Se, which account for their low correlation, can be observed and easily verified in the biplot reported in Figure 1 and confirmed by the correlation coefficient of −0.22. Graphical results for CovSel-LDA, as a projection of samples onto the space spanned by CVs, are shown in Figure 2.

Figure 2 shows that the separation of the three classes of flour, according to the two directions defined by the canonical variates (CVs), was very efficient, resulting in a total correct classification rate of 100% in external validation.

Regardless of the category, the training samples (filled symbols) formed well-defined classes, confirming the high descriptive capability of the calibration model. Similarly, the test set samples (empty symbols), located in the same regions as the training samples of the same class, exhibit similar variability, validating the model’s effectiveness and degree of generalization.

Crickets’ (yellow dots, at negative values of CV1) and mealworms’ (red squares, at positive values of CV1) flours can be distinguished along the first canonical variate (CV1), while samples belonging to the buffalo worm category (green triangles, at negative scores of CV2) can be discriminated from the other two classes along the second component (CV2). Notably, the concentration of selenium (Se), quantified using the present analytical method, is below the LOQ for the buffalo worm class, which exhibits a very narrow dispersion in the CV1–CV2 space. Although the value used for the multivariate analysis was slightly below the LOQ and above the LOD, the classification model indicates that the information provided by selenium concentration may still be helpful in differentiating the considered classes without broadening buffalo warm class dispersion.

Eventually, a forward stepwise-LDA was conducted using the same cross-validation procedure. The selected pool of variables, which demonstrated the best classification ability, included magnesium (Mg), potassium (K), and manganese (Mn), with Mg exhibiting the highest discriminant power across all seven cancelation groups. Among the selected variables, Mg and Mn showed a correlation coefficient of −0.45, while Mg and K had a correlation coefficient of −0.26. The model developed with this set of variables resulted in a classification model with a 100% correct classification rate for all considered classes, both in internal and external validation. In conclusion, Mg was selected for its higher concentration in *T. molitor larvae*, Mn was highest in house crickets compared to the other classes, and K was the highest in the buffalo worm class, as shown in Table 2 and graphically confirmed in Figure 1. These three selected variables, which also demonstrate a good degree of uncorrelation, contributed to a robust and stable linear discrimination model.

## 3. Materials and Methods

### 3.1. Samples

The samples analyzed and classified in this study are obtained from three different species of insects: cricket (*Acheta domesticus*, C), buffalo worms (*Alphitobius diaperinus*, B), and mealworms (*Tenebrio molitor*, M). The crickets and buffalo worms were obtained from their containers already in flour form, while the mealworms were presented in a dried but still whole larval form. Consequently, part of the laboratory procedure was devoted to grinding the mealworms to produce flour. Two grinding methods were utilized: a manual mode using a mortar and pestle and an automatic mode with a batch processing grinder (Tube-Mill control, IKA, Staufen im Breisgau, Germany), operated for 25 s. The reduction of mealworms to flour was carried out using two different methods to assess the potential influence of the grinding process on the ICP-MS analysis data.

According to the indications provided by the producers, crickets used for the preparation of crickets’ flour were in adult form, whereas buffalo worms were in larval form.

Finally, each sample was obtained by performing several replicates on different aliquots, obtained by different sampling from the purchased insects’ flour. A total number of 75 samples (24 samples for cricket flour, 24 for buffalo worm flour, 28 for mealworms) were obtained and considered for chemometric analysis.

### 3.2. ICP-MS Analysis

After the grinding process, all weighed samples were placed in an oven and subjected to a drying process at a constant temperature of 105 °C for 24 h. After drying, the samples were stored in graduated centrifuge tubes for preservation. In total, 50 mg of the dried samples were weighed using the analytical balance and placed in vials to initiate acid digestion. For the acid digestion, 2 mL of HNO_3_ (Sigma-Aldrich, St. Louis, MO, USA, 65%), 5 mL of H_2_O_2_ (Sigma-Aldrich, St. Louis, MO, USA, 30%), and 3 mL of (deionized and demineralized, 18.2 MΩ conductivity) H_2_O Suprapur (Millipore, Bedford, MA, USA), were introduced into the vials. The mineralization process was assisted by a microwave digester (Ethos One, Milestone, Bergamo, Italy). The microwave allowed for the acceleration of the process, minimized the risk of contamination, and improved the quality of the analytical results. After introducing the vials into the instrument, a specific thermal program for mineralization was set: in the first 10 min, the temperature was raised to 180 °C, maintained constant for 1 h, and then returned to room temperature for 1 h. At the end of the mineralization process, the samples were allowed to rest for 24 h to enable the escape of gas bubbles (CO_2_, SO_2_ and NO_X_) resulting from the oxidation of organic matter, which could potentially interfere during the sample introduction phase. Eventually, the samples were brought to volume in 100 mL flasks with H_2_O Suprapur. During this phase, a standard solution of In (Inorganic Ventures, Christiansburg, VA, USA) at a final concentration of 10 µg/L was introduced as an internal standard, useful in estimating and correcting matrix effects. The obtained samples were transferred and sealed in vials for ICP-MS (iCA^TM^ TQe, Thermo Fisher Scientific, Waltham, MA, USA).

The following parameters were set:

- Power: 1500 W
- auxiliary gas (Ar 99.999%, Nippon Gases, Madrid, Spain): 0.8 L/min
- plasma gas: 14 L/min
- nebulizer gas: 1.02 L/min
- extraction lens: −87 V
- focusing lens: 1.45 V
- peristaltic pump: 40 rpm

The proposed method was validated following the Eurachem guidelines [11].

The recovery tests were carried out on fortified samples, which were obtained using the same procedure as for pure samples but with fixed and known aliquots of the analytes at a level of the same order of magnitude as the native analyte concentrations. From the analysis of fortified and corresponding pure samples, recoveries were estimated using the following formula:

(1) $$Recovery\left(\%\right)=\frac{C_{\left(x+a\right)}-C_{x}}{C_{a}}*100$$

where *C*_(*x+a*)_ is the concentration of the analyte after addition, *C_x_* is the analyte concentration before addition, and *C_a_* is the concentration of the added analyte.

Model validation was performed considering two different sample classes, i.e., two slightly different sample matrices (crickets and buffalo worms). Additions were performed before the acid digestion process. To determine the amounts to be added, the average concentration of each element for the two classes was estimated based on data from preliminary analyses conducted in order to set the optimal operating conditions (sample weight, dilutions, calibration range). Working standards solutions with known, increasing concentrations of the target analytes were analyzed for the quantitative analysis. LOD and LOQ were obtained from the calibration curves. By combining information from the analysis of multiple and independent blank samples (n = 6) and the calibration curves, these two parameters were estimated using the following formulas:

(2) $$LOD=\frac{3*SD_{blank}}{a}$$

(3) $$LOD=\frac{10*SD_{blank}}{a}$$

where *SD_blank_* represents the standard deviation associated with experimental measurements of the replicated blank samples, simulating the sample matrix at the same percentage of nitric acid in samples and standards, and *a* represents the slope of the calibration curve.

Working standard solutions were obtained by appropriate dilution of a multi-elemental commercial standard (multi-element reference solution 4 for ICP, TraceCERT^®^, in 10% nitric acid, Merck KGaA, Darmstadt, Germany) and mono-elemental standards (Inorganic Ventures, Christiansburg, VA, USA).

### 3.3. Chemometric Modeling and Validation

Explorative data analysis is a crucial step in chemometrics, as it helps uncover patterns, relationships, and the underlying structure of complex datasets. Principal Component Analysis (PCA) [14,15] is a powerful multivariate technique commonly employed to reduce data dimensionality. By transforming the original variables into orthogonal principal components, PCA facilitates data visualization and highlights dominant trends. Once the data structure is better understood, classification methods can be applied to assign observations to predefined groups. Linear Discriminant Analysis (LDA) [16] is a widely used technique for this purpose, projecting data onto a linear combination of variables (CVs) to maximize the separation between classes.

Forward Stepwise LDA is a commonly used variable selection method in linear discriminant analysis; it is based on an iterative procedure through which relevant predictors can be se selected, improving model interpretability and performance [17]. In this work, the selection of the most discriminant variables was based on the likelihood ratio test statistic (known as Wilks’ statistic) and the F-statistic (which was employed to test the significant decrease in Wilk’s Lambda after variable addition) and was coupled with the cross-validation procedure. Thus, it was possible to evaluate predictive performance in cross-validation as an additional criterion to select the best number of variables to retain in the optimal model. A brief insight into the iterative method is provided below. For each variable, the Wilk’s Lamba is computed as follows:

(4) $$\Lambda =\frac{SS_{W}}{SS_{TOT}}$$

where $SS_{W}$ is the intra-category sum-of-squares, and $SS_{TOT}$ the total sum-of-squares. At this stage, the variable with the lowest lambda value is selected.

The following step is based on the sequential introduction of the variables that lead to a decrease in the multivariate Wilk’s lambda, when more than one variable is involved [18].

(5) $$\Lambda \prime \prime \prime \prime =\frac{\left(I-G\right)\;\left|P\right|}{\left(I-1\right)\left|V\right|}$$

Multivariate Wilk’s lambda is the ratio between the determinants of the pooled variance–covariance matrix, ***P***, and of the generalized variance–covariance matrix, ***V***, normalized for the degrees of freedom (*I* the number of samples, *G* the number of classes).

In each cancelation group, the variable selection was performed as explained before. The classification errors in cross-validation were inspected for each newly inserted variable, which was the main criterion for stopping stepwise addiction. According to each sub-training set’s slightly different characteristics, a more or less different ensemble of variables could be selected in each cancelation group. For the final model, the chosen original variables were the predictors most frequently selected over the cancelation groups, producing the highest classification ability in cross-validation.

In chemometrics, variable selection is essential to ensure robust models, reduce noise, and focus on the most chemically meaningful features. Covariance selection [19] is another approach applied in this study to achieve this, focusing on identifying a subset of variables that best capture the covariance structure of the data. This method helps balance complexity and interpretability, aiding in the development of models that are both accurate and scientifically insightful.

## 4. Conclusions

This study validated a robust and sensitive method for the characterization and classification of insect flours using Inductively Coupled Plasma Mass Spectrometry (ICP-MS) combined with advanced chemometric techniques. By examining the multi-elemental profiles of cricket, buffalo worm, and mealworm flours, the approach demonstrated the potential of ICP-MS for accurate and sensitive detection of elements. Principal Component Analysis (PCA) revealed distinctive patterns in elemental composition among the three insect species, while linear discriminant analysis models using Covariance Selection (CovSel-LDA) and Forward Stepwise Selection achieved 100% classification accuracy in both internal and external validations. Key elements such as magnesium, selenium, manganese, and potassium were highlighted as pivotal for species differentiation. The findings underscore the potential of ICP-MS for routine application in food quality control and regulatory frameworks, particularly in the emerging sector of edible insects as sustainable food sources. The results align with prior research on insect flours’ nutritional and compositional diversity, such as the work by [5], which emphasized the significance of mineral content in differentiating edible insect species. Similarly, this study builds on insights from Benes and collaborators [8] and Foschi et al. [9], extending the applicability of chemometric tools to multi-elemental analysis, thus, enriching the methodological toolkit for food authenticity studies. The study also contributes to the broader dialog on sustainable nutrition and novel food production. Given the increasing demand for alternative protein sources, the ability to rigorously assess and classify insect-based products will enhance consumer confidence and facilitate market expansion. In general, this study represents a significant advancement in the analytical characterization of insect flours by employing ICP-MS combined with chemometric modeling. Unlike existing approaches, which primarily focus on protein or fatty acid profiles, this method underscores the importance of multi-elemental profiling as a discriminatory tool. The selection of critical variables not only ensures robust classification of insect flours but also provides valuable insights into their nutritional and safety attributes. These findings highlight the potential of the approach to address critical gaps in the regulatory and quality control frameworks for novel foods. By bridging the methodological gap between insect and plant-based products, our work lays the foundation for broader applications of ICP-MS in sustainable food innovation. Future research could explore applying this approach to other insect species or flour matrices and investigate the impact of environmental and processing variables on elemental profiles. Such efforts would further cement ICP-MS coupled with chemometrics as a cornerstone methodology in the sustainable food industry. Nevertheless, it can be noted that, despite its effectiveness, the method presents some limitations—for instance, a lengthy sample preparation process and the use of significant quantities of chemicals.

## Figures and Tables

**Table 1:** The isotopes of the analyzed elements, the minimum (LLOQ) and maximum (HLOQ) concentrations in µg/L, defining the extremes of the calibration lines, and the coefficients of determination (R^2^) are reported.

Isotope	LLOQ (µg/L)	HLOQ (µg/L)	R^2^
^10^B	0.5	400	0.9970
^23^Na	150	3000	0.9980
^24^Mg	75	3000	0.9930
^27^Al	0.2	160	0.9980
^31^P	300	8000	0.9980
^39^K	300	8000	0.9999
^44^Ca	50	1000	0.9960
^52^Cr	0.1	80	0.9980
^55^Mn	0.2	40	0.9980
^57^Fe	2	400	0.9990
^59^Co	0.05	40	0.9990
^60^Ni	0.1	80	0.9990
^64^Zn	0.5	400	0.9999
^65^Cu	0.1	80	0.9999
^80^Se	0.5	400	0.9990
^88^Sr	0.25	20	0.9810
^98^Mo	0.25	20	0.9999
^111^Cd	0.05	40	0.9999
^138^Ba	0.2	160	0.9999

**Table 2:** Validation parameters of the method for each analyzed isotope and mean concentrations of the element in the different classes of flours (crickets (C) and buffalo worms (B) and 28 samples for mealworms (W)).

Parameters	^10^B	^23^Na	^24^Mg	^27^Al	^31^P
RSD (%)	X	0.4	1	6	1
RecoveryB (%)	X	99	96	103	105
RecoveryG (%)	X	99	96	104	93
mean B class (ppm)	<LOD	1760	550	220	7200
mean G class (ppm)	<LOD	2900	464	260	8400
mean W class (ppm)	<LOD	560	1160	80	6800
LOD (ppm)	5	3	4.5	0.8	0.2
LOQ (ppm)	17	9	15	3	0.7
**Parameters**	**^39^** **K**	**^44^** **Ca**	**^52^** **Cr**	**^55^** **Mn**	**^56^** **Fe**
RSD (%)	1	1	6	2	3
RecoveryB (%)	95	102	93	95	96
RecoveryG (%)	107	109	93	101	102
mean Bclass (ppm)	11400	680	0.4	6.6	52
mean Gclass (ppm)	10000	1220	0.2	46.6	60
mean Wclass (ppm)	9800	380	<LOQ	13.4	56
LOD (ppm)	23	120	0.02	0.3	0.0002
LOQ (ppm)	73	393	0.07	1	0.0007
**Parameters**	**^57^** **Fe**	**^59^** **Co**	**^60^** **Ni**	**^64^** **Zn**	**^65^** **Cu**
RSD (%)	3	4	7	3	3
RecoveryB (%)	101	120	101	103	100
RecoveryG (%)	101	120	102	107	106
mean Bclass (ppm)	68	0.06	0.6	130	38
mean Gclass (ppm)	80	0.04	0.6	320	38
mean Wclass (ppm)	78	0.06	0.6	140	22
LOD (ppm)	0.004	0.004	0.15	3	0.07
LOQ (ppm)	0.013	0.013	0.50	9	0.2
**Parameters**	**^80^** **Se**	**^88^** **Sr**	**^98^** **Mo**	**^111^** **Cd**	**^138^** **Ba**
RSD (%)	4	2	2	1	3
RecoveryB (%)	X	X	95	91	98
RecoveryG (%)	93	98	95	92	96
mean Bclass (ppm)	<LOQ	<LOQ	0.78	0.032	0.52
mean Gclass (ppm)	0.54	4.2	0.8	0.032	2.88
mean Wclass (ppm)	0.28	3.24	1.42	0.048	3.3
LOD (ppm)	0.07	0.5	0.04	0.01	0.04
LOQ (ppm)	0.25	2	0.13	0.03	0.12

## Data Availability

Data will be available on request to the corresponding author.
